# Fungal–plant interaction: a pathogenic relationship between *Ganoderma segmentatum* sp. nov. and *Vachellia nilotica*

**DOI:** 10.3389/fmicb.2024.1411264

**Published:** 2024-07-24

**Authors:** Aisha Umar, Wanlan Yuan, Junxing Lu, Fuad Ameen

**Affiliations:** ^1^Chongqing Key Laboratory of Plant Environmental Adaptations, College of Life Science, Chongqing Normal University, Chongqing, China; ^2^Institute of Botany, University of the Punjab, Lahore, Pakistan; ^3^Department of Botany and Microbiology, College of Science, King Saud University, Riyadh, Saudi Arabia

**Keywords:** laccate fungi, paleotropic species, basal rot, ITS rDNA, Ganodermataceae, laccase

## Abstract

The diversity of *Ganoderma* remains largely unexplored, with little information available due to fungiphobia and the morphological plasticity of the genus. To address this gap, an ongoing study aims to collect and identify species with this genus using nuclear ribosomal DNA regions called the “Internal Transcribed Spacer” (ITS1-5.8S-ITS2 = ITS). In this study, a new species, *Ganoderma segmentatum* sp. nov., was discovered on the dead tree trunk of the medicinal plant, *Vachellia nilotica*. The species was identified through a combination of morpho-anatomical characteristics and phylogenetic analyses. This new species was closely related to *Ganoderma multipileum, G. mizoramense*, and *G. steyaertanum*, with a 99% bootstrap value, forming a distinct branch in the phylogenetic tree. Morphologically, *G. segmentatum* can be distinguished by its frill-like appearance on the margin of basidiome. Wilt or basal stem rot, a serious disease of trees caused by *Ganoderma* species and *V. nilotica*, is brutally affected by this disease, resulting in substantial losses in health and productivity. This *Ganoderma* species severely damages *V. nilotica* through deep mycelial penetration in the upper and basal stems of the host species. Pathogenic observational descriptions of *G. segmentatum* on dead tree trunks showed the exudation of viscous reddish-brown fluid from the basal stem portion, which gradually extended upward. Symptoms of this disease include decay, stem discoloration, leaf drooping, and eventual death, which severely damaged the medicinal tree of *V. nilotica*.

## 1 Introduction

The members of the genus *Ganoderma* P. Karst. (Basidiomycota, Polyporales) can be found all over the world and play a diverse role in forest ecosystems (Coetzee et al., 2015; Sun et al., 2022). The tropical diversity of *Ganoderma* is not completely clear, as shown by the current number of novelties (Wang et al., 2009; Xing et al., 2018; Cabarroi-Hernández et al., 2019; Luangharn et al., 2019). The synonymization in the genus *Ganoderma* indicates that the present nomenclatural situation is unsatisfactory and confused, and its improvement needs continuous endeavor (Hong and Jung, 2004; Wang et al., 2009; Fryssouli et al., 2020). *Ganoderma* has been investigated at macro- and micro-levels, and some existing records are purely based on morphological descriptions. The main morpho-anatomical characteristics of *Ganoderma* species are shelf-like basidiomata, laccate appearance, and a maroon-brown color (Ahmad, 1956; Steyaert, 1972; Irshad et al., 2012; Fakhar-ud-Din and Mukhtar, 2019). Unfortunately, no DNA sequences are available for a few records, and there is an urgent need to use molecular methods to study the *Ganoderma* species (Umar et al., 2021a).

Molecular phylogeny provides interesting progress in this genus (Volk, 1992; Moncalvo, 2000; Hong and Jung, 2004). Most sequences of ITS rDNA have been analyzed to delimit and resolve the confusion surrounding this species in *Ganoderma* complexes (Hong and Jung, 2004; Cabarroi-Hernández et al., 2019). The internal transcribed spacer (ITS: ITS1-5.8S-ITS2) shows maximum efficiency in solving the problems regarding species identification among *Ganoderma* taxon (Fryssouli et al., 2020). ITS presents a clear barcoding gap to sort out the morphological and biological species concepts; therefore, it can be used for identification (Badotti et al., 2017).

*Ganoderma*, a hazardous white rot fungus present on living trees, possesses cell wall-degrading enzymes to degrade major wood components such as lignin, cellulose, and hemicellulose (Schwarze et al., 2000). Many *Ganoderma* species are pathogenic, causing butt and stem rot diseases of woody plants and crops (Moncalvo, 2000; Elliott et al., 2001; Sahebi et al., 2017). Saprophytic *Ganoderma* species are highly ligninolytic in nature due to white rot (Volk, 1992). In natural ecosystems, laccase plays an important role in lignin production and degradation in plants. Laccases have garnered multiple industrial interests, e.g., xenobiotic bioremediation and detoxification of phenolics (Wang et al., 2015). This enzyme has been presented in many *Ganoderma* species and is associated with diverse biological events, especially in the life cycle of white-rotter's mycelium development, pigmentation, sporulation, and fruiting body formation (Jin et al., 2018). *Ganoderma* species are emerging as major fungal pathogens. Laccases of these species have been assigned other biological functions, e.g., plant pathogenesis, lignin degradation, and provides understanding of how a fungal saprophyte becomes a dangerous pathogen. It is an intrinsic attribute for an opportunistic pathogen to realize that its regulatory pathways have evolved under environmental rather than host pressure. Nevertheless, its production pathway defines the pathogen's ability to cause host damage because evolution allows for appropriate survival in the environment and pathogenicity in the host plant (Zhu and Williamson, 2004).

Higher plants assimilate laccases in lignin biosynthesis and polymerization (a structural compound), which further facilitate plant development and defense of plant tissues in response to pathogenic species (Arregui et al., 2019; Westrick et al., 2024). Interestingly, *Ganoderma*, a wood rotter, secretes laccase during lignin degradation, in which terminal phenolic lignin is directly oxidized by laccases (Westrick et al., 2024).

Many diseases are caused by different pathogens in multiple crops and trees, e.g., *Ganoderma* sp., which leads to basal and upper stem rot (Fee, 2011). The genus *Ganoderma* significantly minimizes the yield of medicinal plants, and the world economy experiences a huge product loss, if we fail to protect the medicinal plants (Corley and Tinker, 2008). Degradation by *Ganoderma* slowly leads to the decay of tree trunks, a process where lignin and cellulose are diminished with the passage of time. Trunks of trees die due to the infectious pathogenic action of *Ganoderma*, where lignin and cellulose of the lower bole or root flare become decayed (Sinclair and Lyon, 2005). Proteins and cell wall-degrading enzymes are biological macromolecules involved in fungal life activities (Wu et al., 2017). Fungal attack directly exposed the spongy cellulose by decomposition of lignin, and white rot appeared on the plant's woody tissues (Paterson et al., 2009). The disease of rot starts with the penetration of fungal mycelia into the tree's roots, which is sporadic toward the shoot, and finally, the trunk stands collapse (Rees et al., 2009).

*Vachellia nilotica is* a tree of 5–20 m long, fissured bark with a dense spheric crown. The stems and branches are usually dark to black in color. This tree has thin, straight, light gray spines in axillary pairs without thorns. Leaves are bipinnate, with pairs of pinnulae, tomentose, and rachis, with a gland at the bottom of the last pair of pinnulae. Flowers are globulous (1.2–1.5 cm dia.), bright golden-yellow in color, and whorly on 2–3 cm long peduncles located at the ends of branches (Wardill et al., 2005). The present study was conducted to analyze the morpho-anatomical characters of basidiomata and the ITS sequence phylogeny of new *Ganoderma* specimens, which act as a major pathogen of *V. nilotica*. This species leads to massive economic losses by threatening the medicinal host plants with basal rot diseases.

## 2 Materials and methods

### 2.1 Specimen collection and morphological examination

The *Ganoderma* specimens were collected in 2019 from the University of the Punjab, New Campus, Lahore, Pakistan (under the authority letter of the Director, Institute of Botany, University of the Punjab). The average annual rainfall is approximately 607 mm, and the temperature is 24°C at this site. This area is covered by *Vachellia nilotica*. Basidiomata were found at the bases of the live tree trunks of *V. nilotica* trees, usually after the onset of the rainy season in early summer. We observed microscopic structures from cross-sections of the dried basidiomata in KOH (5%), stained them with Congo red (1%), and viewed them under an MX4300H compound light microscope (Meiji Techo Co., Ltd., Japan). Drawings were made with the aid of a drawing tube. Data on anatomical features were recorded from at least 30 measurements at a magnification of 1000X. Basidiospore measurements were presented as length × width (Nagy et al., 2010). Basidiospores were measured without taking into account the apiculus when they were not shrunk. The morphological descriptions of the microscopic features are in part following the study by Cabarroi-Hernández et al. (2019). Color terms in parentheses were recorded by following Kornerup and Wanscher (1975).

### 2.2 DNA sequencing and phylogenetic analyses

Genomic DNA was extracted from dried specimens using the modified cetyltrimethylammonium bromide (CTAB) procedure (Doyle and Doyle, 1987). ITS (ITS1+5.8S+ITS2 rDNA) was amplified with polymerase chain reaction (PCR) using the primers ITS1 and ITS2 (White et al., 1990). Reaction mixtures (20 μL) contained 0.5 μL DNA template, 8.5 mL of distilled water, 0.5 μL of each primer, and 10 mL PCR mix [DreamTaqGreen PCR Master Mix (2 X), Fermentas]. PCR cycling conditions were 35 cycles of 95°C for 30 s, 52°C for 30 s, and 72°C for 1 min, followed by a final extension at 72°C for 10 min. Sequencing was conducted using the same PCR primers. New sequences were edited using MEGA v. 10.0 (Kumar et al., 2016) and submitted to GenBank under accession numbers MZ666127 and MZ666128.

The newly generated sequences were used to search for the most similar sequences deposited in GenBank using the BLASTn tool. Sequences were aligned using the MAFFT online version (Katoh et al., 2019) and manually adjusted using MEGA v. 10.0 (Tamura et al., 2011). A phylogenetic analysis was conducted via maximum likelihood (ML) and Bayesian inference (BI). ML analyses were performed using RAxML-HPC Blackbox version 8.2.10 (Stamatakis, 2014) implemented on the CIPRES Science Gateway (Miller et al., 2010), with an estimated proportion of invariable sites GTRGAMMA+I and branch support evaluated by 1,000 bootstrap replicates. Bayesian inference was carried out in MrBayes v. 3.2.2 (Ronquist et al., 2012). The best substitution model for tree reconstruction was estimated by both the Akaike information criterion and the Bayesian information criterion jModelTest 2.0 (Darriba et al., 2012). Four Markov chain Monte Carlo chains were run simultaneously, starting from random trees for 2,000,000 generations and sampling every 100th generation. After discarding the first 25% of trees as burn-in phase, a consensus Bayesian tree and Bayesian posterior probabilities (BPP) were determined based on all remaining trees. A BPP above 0.90 was considered a significant value. Trees were visualized and further edited in Tree Graph 2 (Stöver and Müller, 2010).

### 2.3 Qualitative analysis and absorption spectra

Malt Extract Agar (MEA) media was prepared by the addition (g/L) of MEA 7, Agar 10, K_2_HPO_4_ 0.5, KH_2_PO_4_ 0.5, MgSO_4_·7H_2_O 0.5, Peptone 2.5, and Glucose 15 at pH 5.0. Culture media was sterilized (for 25 min at 121°C) and then autoclaved. The autoclaved MEA was augmented with 0.02% guaiacol for typical laccase (phenolic) and 0.04% veratryl alcohol (non-phenolic) for atypical laccase. Chromogen was added to evaluate the atypical and the typical laccase-producing abilities of *Ganoderma* mycelium. The replicates were incubated at 30°C for 5 days.

Submerged culture broth was designed for peak absorption of laccase in shake flasks. Submerged nutrients broth (g/L), composed of yeast extract 5 g, starch 1 g, MgSO_4_·7H_2_O 0.5 g, NaCl 0.5 g, FeSO_4_·7H_2_O 0.5 g, CaCl_2_ 0.5 g, and ZnSO_4_ 0.02 g, were taken in shake flasks for the growth of mycelium and incubated at 27± 2°C in static condition for 5 days. The pH range of 8.0 to 11 for blue laccase and 4 to 12 for white laccase was selected. Afterward, absorbance was monitored at 280, 330, 470, and 605 nm (3 min) using a UV-Vis spectrophotometer (Xing et al., 2018). Three replicates were designed for accuracy in the results.

### 2.4 Statistical analysis

The collected data from various parameters was analyzed and represented by means ± standard deviation (SD). Statistical analysis was calculated using SPSS 18.0 software.

## 3 Results

### 3.1 Phylogenetic analyses

**Figure 1:**
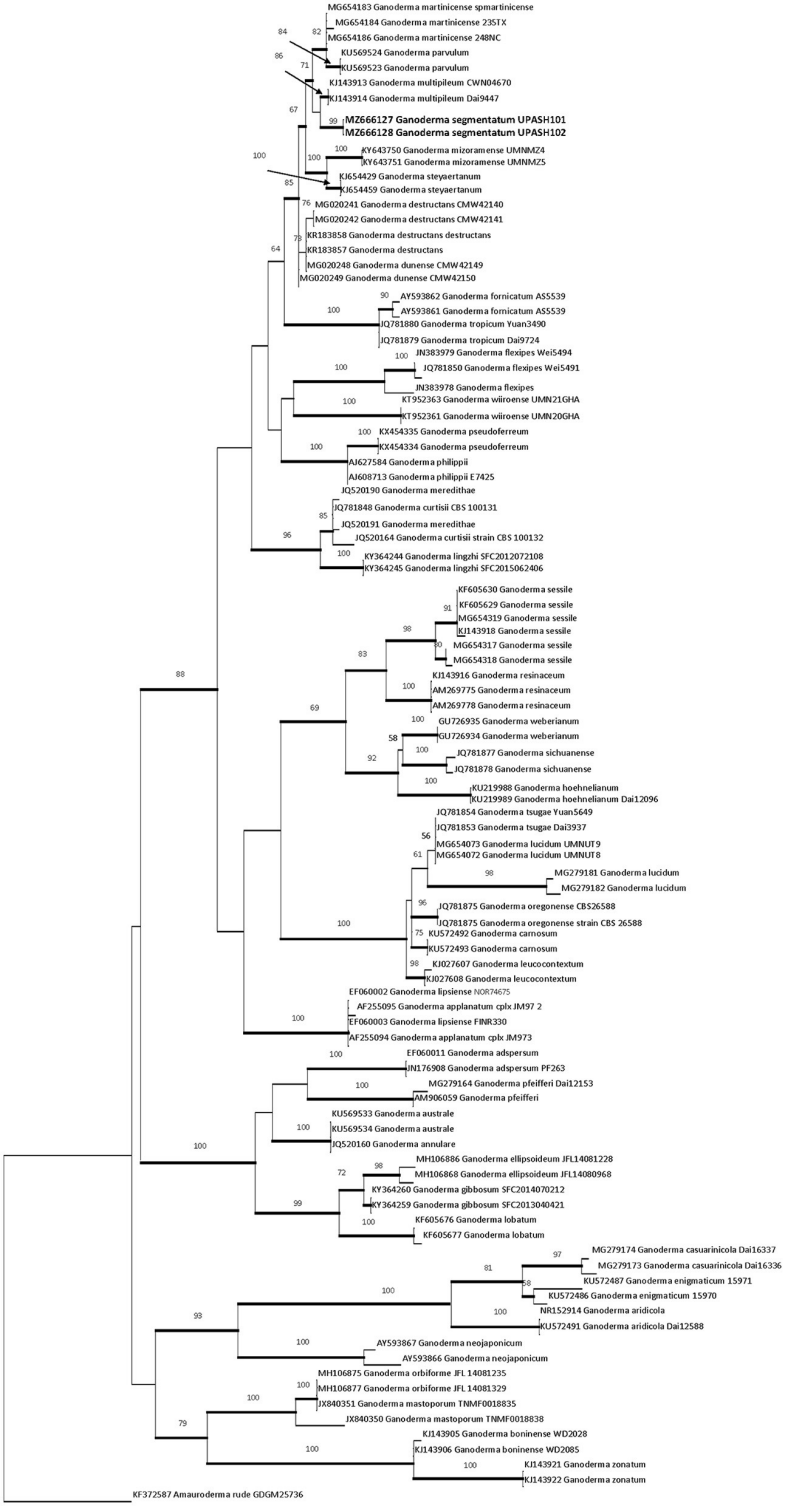
RaXmL tree reconstructed based on ITS rDNA sequences showing the phylogenetic placement of *G. segmentatum* sp. nov. Maximum likelihood bootstrap (MLB) values higher than 50% (based on 1,000 replicates) are displayed at the nodes, and thick lines represent Bayesian posterior probabilities (BPP) >0.95. The tree is rooted in *Amauroderma rude* (KF372587). The sequences in bold are from the new species.

The two ITS rDNA sequences (MZ666127 and MZ666128) of the new species had 0.5% site differences. The phylogenetic tree of *G. segmentatum* sp. nov. was closely matrixed to *G. multipileum, G. mizoramense, and G. steyaertanum* B. J. Smith and K. Sivasithamparam with a bootstrap value of 99%. The ITS dataset included 98 nucleotide sequences of *Ganoderma* species, and one sequence of *Amauroderma* (*A. rude* KF372587) was chosen as an outgroup in this tree. There were 697 bp in the dataset. The nucleotide frequencies were A = 22.21%, T/U = 29.35%, C = 23.56%, and G = 24.87%, and the maximum log likelihood was *InL*-4430,914. The best model, GTR+G+I, selected by jModelTest, was used for BI. Both ML and BI analyses resulted in trees of identical topology, and the differences mostly supported the nodes. The ML tree is shown in Figure 1.

### 3.2 Taxonomy

**Figure 2:**
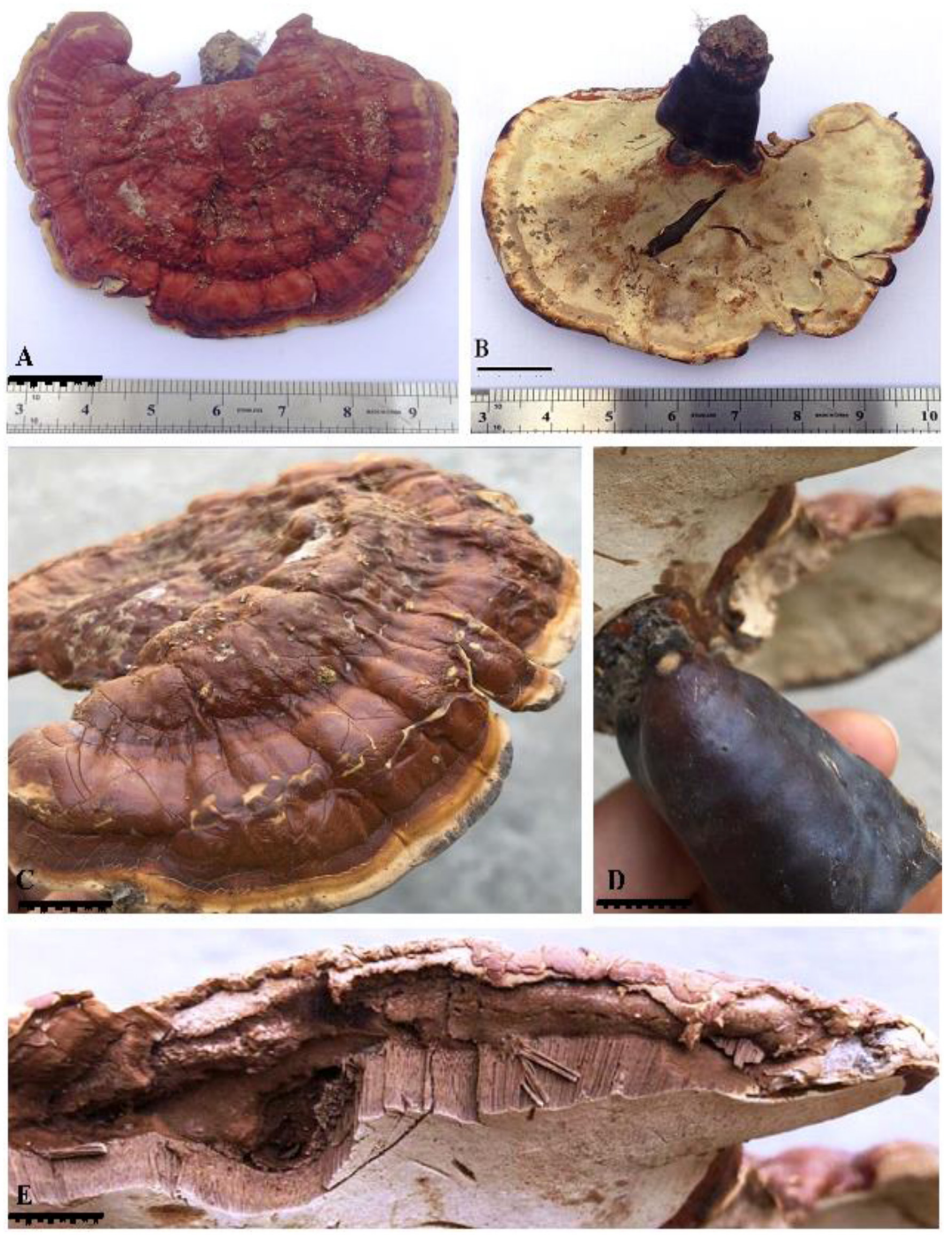
*G. segmentatum* sp. nov. **(A)** basidiome upper surface with a frill-like margin and laccate appearance. **(B)** milky white lower pore surface. **(C)** side view of the basidiome with thin margins. **(D)** caccate dark maroon brown stipe. **(E)** contextum in layers form and longitudinal tubes below the contextum [scale bars: **(A–D)** = 3 cm, **(E)** =0.5 mm].

**Figure 3:**
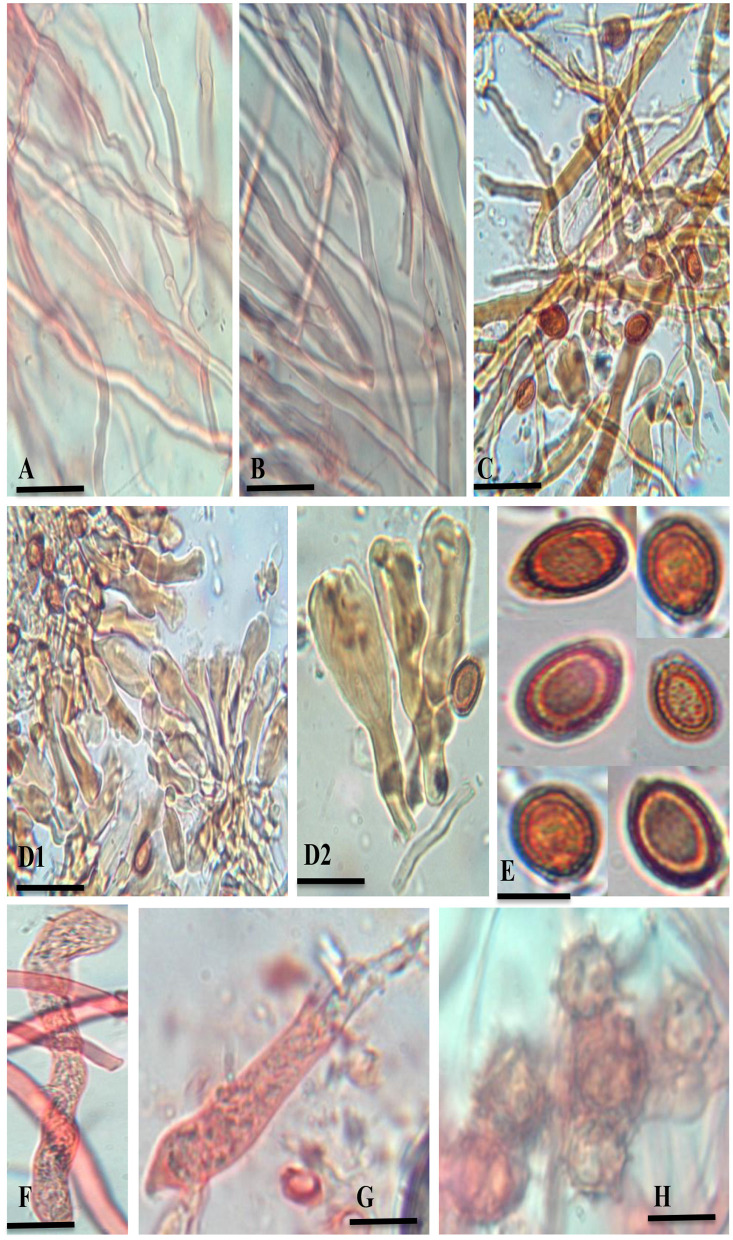
*G. segmentatum* sp. nov. **(A)** generative hyphae (clamp connections indicated by an arrow). **(B)** skeletal hyphae. **(C)** binding hyphae with multiple branches. **(D**1, **D**2**)** crustohymeniderm cells/cuticle cells taken from basidiomata. **(E)** broadly ellipsoid, bitunicate, and coarsely echinulated basidiospores. **(F, G)** basidia. **(H)** chlamydospores [scale bars: **(A–C)** =5 μm, **(D–G)** =10 μm].

**Figure 4:**
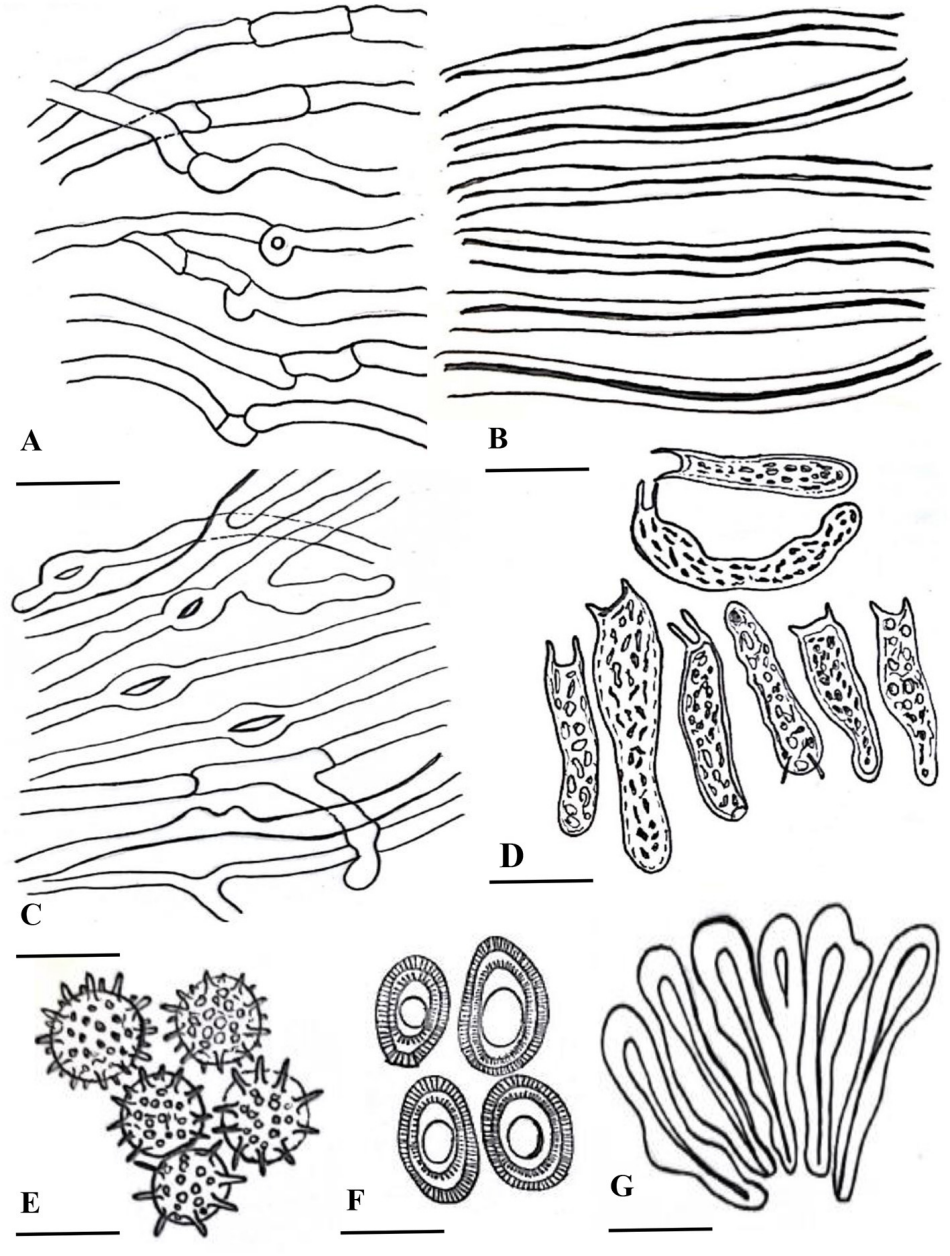
Line drawing of anatomical characters of *G. segmentatum*. **(A)** cenerative hyphae. **(B)** skeletal hyphae. **(C)** binding hyphae. **(D)** basidia. **(E)** chlamydospores. **(F)** basidiospores. **(G)** crustohymeniderm cells [scale bars: **(A–C)** =5 μm, **(D–G)** = 10 μm].

***Ganoderma segmentatum*** A. Umar (Figures 2–4)

MycoBank: MB840865

*Ganoderma segmentatum* is morphologically characterized by annual, dimidiate, stipitate, and brick-red-colored laccate basidiome with a thin, light brown margin. A context without resinous melanoid bands was observed in this species.

**Holotype**: PAKISTAN. Punjab Province: Lahore, New Campus, University of Punjab (31.4981°N 73.3044°E), elevation 217 m a.s.l., attached to the trunk of the living tree of *Vachellia nilotica*, 22 June 2018, Aisha Umar (holotype UPASH101). GenBank: ITS = MZ666127.

**Etymology**: The species epithet “*segmentatum*” indicates the frill-like appearance at the pileus margin.

**Description: “Basidiomata** annual, solitary or in group, rigid, dull laccate, stipitate, convex, consistency corky-woody; **Pileus** 17–18 × 13–13.5 cm, applanate, non-imbricate, dimidiate to flabelliform, laccate, plano convex, hard, shiny upper surface, rugose to verrucose, thin crusted, conspicuously concentrically sulcate to groovy, particularly with frill-like appearance near the margin and around the whole basidiomata; purplish red (14A8), earth colored (5F3), brick red; **Stipe** 5.5–5.5 × 3.5–3.7 cm, pleuropode, acentric, stout, cylindrical to flat dorso-lateral, dark brown (8F7–6F8) thus darker than the pileus, woody hard, strongly laccate with a thick crust; **Margin** 0.3–0.5 mm thick, obtuse, entire, few undulations, usually lighter than the rest of the pileus, dull yellow (4A8) to brown (5E8) when fresh, and later cream (4A3) to orange (5A4) in dry condition; **Stipe** 5.5–5.5 × 3.5–3.7 cm, pleuropode, acentric, stout, cylindrical to flat dorso-lateral, dark brown (8F7–6F8) thus darker than the pileus, woody hard, strongly laccate with a thick crust; **Pore surface** at first cream (4A3) becomes brown (6E8) to dark brown (6F8) when old, pores almost invisible to the naked eye, subcircular to circular, dissepiments entire and thick, sterile margin concolorous; **Tubes** 1.1–1.2 cm deep, non-stratified, grayish brown (6E3) with earth-colored (5F3) walls, strongly contrasting with the pore mouth and the context; **Context:** 0.9–1.2 cm thick, dry, azonate in both pileus and stipe, loose fibrils, rusty brown (6E8) to dark brown (6F8), hard, non-melanoid, separated from the crust by a yellowish orange (4A7) thin line; **Hyphal system trimitic:** generative hyphae hyaline, thin-walled, septate, with clamp connection, abundant in the dissepiments; somatic hyphae composed of skeletal and binding hyphae; skeletal hyphae thick-walled to solid in the trama; frequently branched, yellowish (2A3), dichotomous to arboriform with thick stalks gradually tapering in the context; and cuticle yellowish (2A3) to brownish (6D6), with thick branches shorter than in the trama; binding hyphae thick-walled, light brown (5D5), randomly bulbous (from middle or near the branch), branched slender, and acutes; **Pileipellis:** composed of a palisade of vertical club-shaped to broadly clavate, pale yellow, yellowish-brown (5E8) to pale dark brownish (6E8) apices, 38.5–55.7 × 12.2–14.4 μm, smooth, thick-walled to solid, with scant lumen, largely stalked with peduncles continuing as hyaline thick-walled hyphae; **Basidia** 10.5–20.2 × 2.2–2.7 μm, fusiform to clavate, mostly two-spored with acute sterigmata, thin-walled, content with fine to coarse granular oily content; **Basidiospores** 8.5–9.6 × 5.2–6.7 μm (average L = 8.84 μm, W = 5.78 μm, Q = L/W = 1.52), broadly ellipsoid, bitunicate, exospore smooth, endospore coarsely echinulate, with turgid vesicular appendix, guttulated, spore print light brown (6D8); **Chlamydospores** 2.4–2.9 × 2.3–2.6 μm, numerous, round, thick-walled, ornamented with long pillars, hyaline, yellowish (2A3) to reddish brown (8E7).

**Another specimen examined:** PAKISTAN. PUNJAB PROVINCE: Lahore, New Campus, University of Punjab (31.4981° N 73.3044° E), elevation 217 m a.s.l., attached to a dead tree trunk of *Vachellia nilotica*, 25 July 2019, Aisha Umar (isotype UPASH102). GenBank: ITS = MZ666128.

### 3.3 Plant-pathogenic interaction

**Figure 5:**
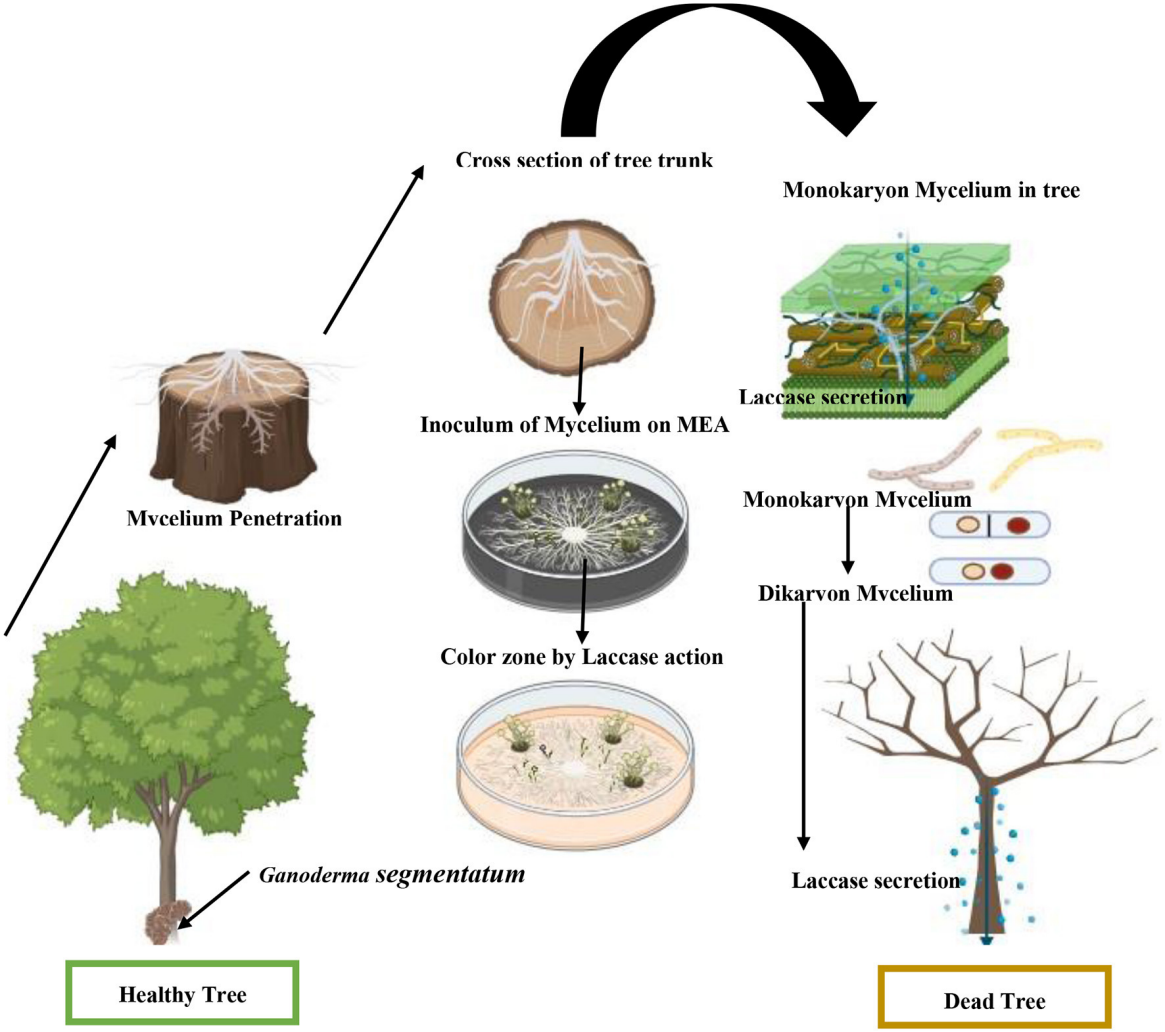
Schematic presentation of stem root causes the decay of *V. nilotica* by *G. segmentatum* sp. nov.

*Ganoderma segmentatum* is reproduced by vegetative mycelia and sexually by spores. Basidiospores are believed to be the source of the inoculum. Spores germinated and formed the monokaryotic vegetative mycelia, which are saprophytically grown in the plant trunk. Dikaryotic mycelium was seen via migration and the nuclear exchange of two different hyphae. The dikaryotic mycelium of *Ganoderma* species caused a deep and speedy invasion within the plant host. The dikaryotic mycelia formed the large basidiomata under suitable environmental conditions. Basidiomata is a multicellular reproductive body where karyogamy and meiotic spores lead to the completion of the sexual phase (Figure 5). The tetrapolar mating system in sexual reproduction promoted the diversity of genetic content in the same plantation of the genus *Ganoderma*, leading to population dynamics. This is the primary cause of inefficient disease management. Basal rot management is a big challenge, because the infective dikaryotic mycelium continued the process of penetration into a healthy tree trunk and produced new basidiospores.

Colonization of *G. segmentatum* with *V. nilotica* was observed through contact between healthy and diseased roots. The colonization started when the *Ganoderma* species found a wound for penetration (Figure 5). *G. segmentatum* initially exhibited the symptoms of reddish-brown viscous fluid exudated from the basal stem portion, which gradually extended upward in direction. In severe cases, the basal portion of the stem decayed completely, and sporophores appeared at the base of the trunk. Brutally rot roots, decay and discoloration of the stem, drooping of all leaves, browning of branches, and death of the plant are the characteristic symptoms of this disease.

The growing patches of BSR infection covered the healthy tree species with the passage of time. The specialized reproductive basidiospores of *Ganoderma* play an important role in the maintenance of the sexual cycle. *Ganoderma segmentatum* is characterized by broadly ellipsoid, bitunicate, smooth exospores, endospores that are coarsely echinulated, turgid vesicular appendix, and guttulated basidiospores typically grown in the trunk. Similarly, other white-rot fungal species degrade the wood lignin component during pathogenic action. Basidiomata on the basal stem badly infected the *Vachellia nilotica* tree. Symptoms of basal stem rot disease on *V. nilotica* were the collapsing of lower leaves and leaves hanging downward from the point of attachment, and finally, the trunk falling down due to stem decay. *Ganoderma segmentatum*, as a pathogen, released cell wall-degrading laccase to soften and loosen the host cell wall made up of lignin. The fungal and its laccase facilitated the softening and deep penetration of needle-like microhyphae in the stem of *V. nilotica*. Microhyphae with extracellular matrix degraded the cellulose components, leading to minute cell wall cracks. This pathogenic interaction of mycelium with living cells and tissues also facilitated the continuous supply of nutrients. Microscopic examination of infected *V. nilotica* revealed the biotrophic nutrition provided by *G. segmentatum* during colonization. Needle-like fungal structures puncture the healthy plant cells. Embedded thick and blackish-gray lines were also observed in the host tree.

### 3.4 Cell wall degrading enzyme

**Figure 6:**
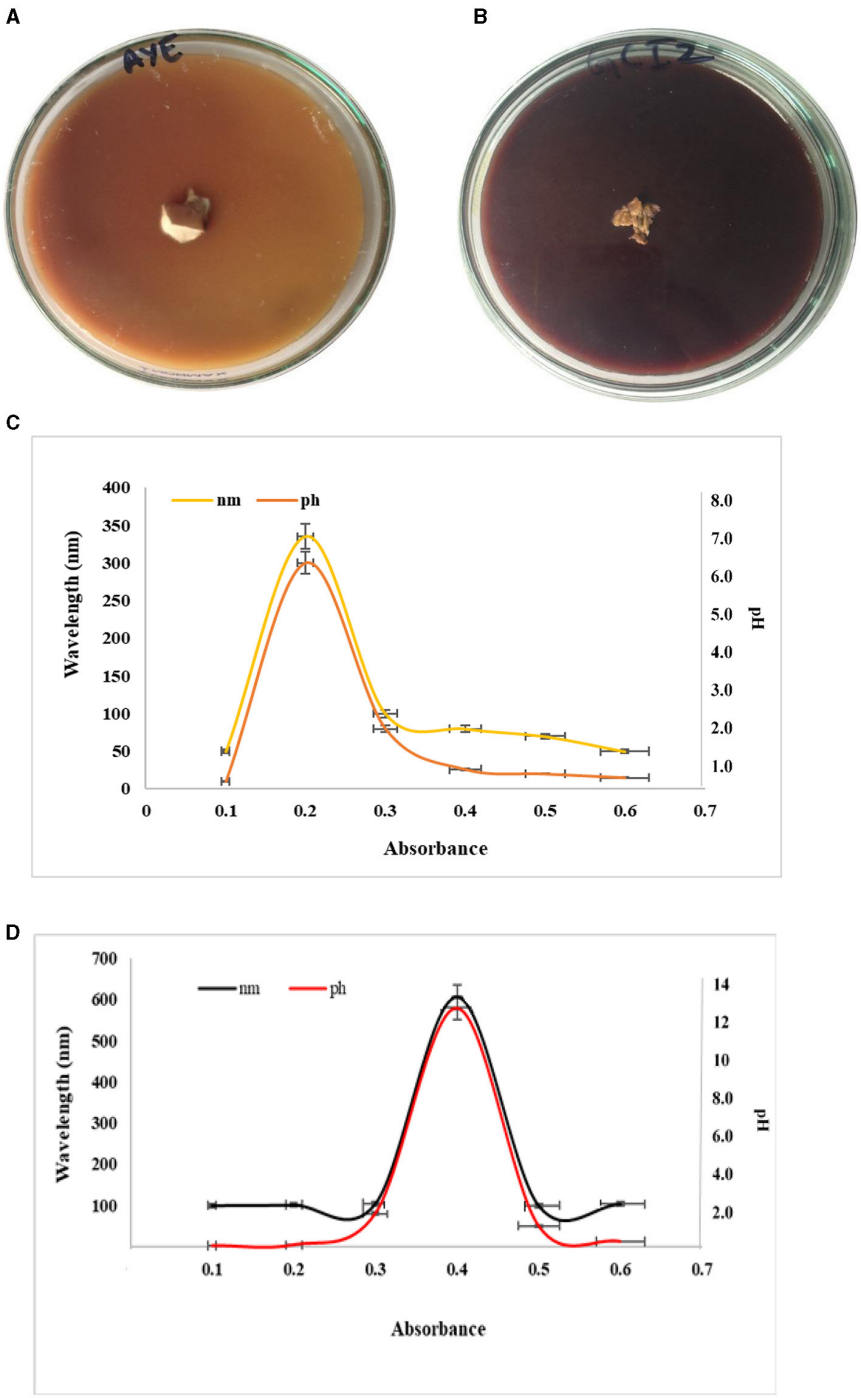
Preliminary plate test for laccase **(A)** veratryl alcohol, **(B)** guaiacol, **(C)** absorbance of laccase, and **(D)** laccase at a particular pH.

The laccase in this study was detected in the preliminary test. The engraved mycelium was taken exactly below side of the fruiting body grown on the tree trunk of *V. nilotica*. Trunk-penetrated mycelia were inoculated on an MEA plate augmented with veratryl alcohol. White laccase transformed the veratryl alcohol into a brown color by oxidation (Figure 6A). Blue laccase formed the reddish-brown oxidation zone on the agar plate after oxidation of guaiacol, indicating the ability of *Ganoderma* sp. to release the laccase (Figure 6B). It is well known that blue laccases generally react with organic substrates in the presence of mediators (guaiacol), while yellow laccases perform the same act without any mediator molecules. The laccase of *Ganoderma* species oxidizes the non-phenolic lignin compounds, which cannot be oxidized by the laccase alone. Thus, the oxidation of lignin was dependent on the presence of primary laccase substrates (mediators). In this study, typical blue laccase exhibited a reddish-brown color by reaction with chromogen, while white or yellow laccase did not exhibit any color with guaiacol.

Laccase secreted by mycelium taken from the lower side of the fruiting body exhibited a peak at 340 nm at a pH of 6.5 (Figure 6C). The absorption peak was 600 nm at a pH of 12 for laccase in submerged broth (Figure 6D). The results indicated from the peak that laccase broke down the trunk and caused basal stem rot via the production of white laccase. The peak observed for laccase in the submerged broth indicated blue laccase.

White/yellow laccase had an absorption peak at 330 nm to 400 nm, but no peak was observed at 605 to 610 nm. Yellow laccase can be reduced artificially into blue laccase but it does not exhibit absorption at 600 nm. Conversion of laccase from blue to yellow occurred by the reduction of the type I Cu site via aromatic products or the binding of specific amino acids to the enzyme polypeptide formed during lignin degradation. The reason behind the colorlessness of white laccase is the change in the valence state of the copper ion (Cu^2+^). White laccase is also considered a member of the laccase family because its primary structure is identical to the known laccase, which uses oxygen as an oxidative agent.

## 4 Discussion

In the ITS phylogeny, the new species clustered together with *G. multipileum, G. martinicense, G. mizoramense*, and *G. destructans* M.P.A. Coetzee, Marinc, and M. J. Wingf and *G. steyaertanum* in a highly supported clade (99%). The global meta-analysis of ITS rDNA *Ganoderma* sequences (Fryssouli et al., 2020) resolved all five species with a maximum supported (72% ML, 0.97 BPP) lineage. Fryssouli et al. (2020) used the threshold of interspecific values for ITS similarities (≤ 98%), and genetic distance (≥ 0.015) effectively applied to the 21 putatively new phylospecies. The widely adopted thresholds for separating the species in Basidiomycota are < 97% to 98% for ITS sequence similarity, while *p*-values are > 0.010–0.020 for genetic distances (Zervakis et al., 2019). Therefore, ITS-based phylogenies are helpful in addressing taxonomic issues (in conjunction with other criteria like distinct morpho-anatomical characters).

The present study introduced *G. segmentatum* as a new member of the genus *Ganoderma*. It also substantiated the reliability of our ITS phylogeny in resolving *Ganoderma* species in this clade. The six species in the lineage can be roughly characterized by the geographical origin of their specimens. The specimens of *G. destructans* originated from South Africa (Darriba et al., 2012; Coetzee et al., 2015). *G. martinicense* is reported from the USA, Mexico, Cuba, Martinique, Colombia, Brazil, and Argentina (Darriba et al., 2012). *G. steyaertanum* is distributed in Indonesia and Australia, while *G. multipileum is* described as including the specimens formerly assigned to tropical Asian *Ganoderma lucidum* (Stöver and Müller, 2010). As shown by the internal clade resolution, *G. multipileum* had a strong phylogenetic relationship (86% ML, 0.95 BPP) with *G. segmentatum*, which was resolved in the sister position. Both species form a sister subclade with *G. martinicense*. Based on present knowledge, *G. multipileum* and *G. segmentatum* have the same geographical origin, especially *G. multipileum*, which is known from Pakistan. Besides the phylogenetic separation, the two species can be well differentiated by their morphological features. Basidiomata of both species are usually laterally stipitate, while in *G. segmentatum*, they can be concrete, with pilei often growing together from the lower pilei in *G. multipileum* (Wang et al., 2009). A group of laccate pilei in *G. multipileum* overlap, while our new species is less laccate and solitary, appearing on a stem. The basidiomata is maroon-brown in *G. multipileum* and brick-red in *G. segmentatum*. In addition, the imbricate and concrete basidiomata of *G. multipileum* may reach a large size (up to 36 cm long and 54 cm wide) (Wang et al., 2009). Moreover, the pileial surface of *G. multipileum* showed a range of colors from orange-yellow (4A7) to orange-red (8A8) to brown-red (8C8) and was radially striate (Wang et al., 2009) compared to the sulcate concentrically zonate margin of *G. segmentatum*.

The most distinguishing features are the size and shape of the basidiospore in the genus *Ganoderma* (Steyaert, 1972; Kirk et al., 2008; Torres-Torres and Guzmán-Dávalos, 2012; Umar et al., 2021b). The spore size of *G. multipileum* is larger (8.0–13.2 × 5.5–7.4 μm) than that of our species. On an ITS basis, 26 additional nucleotides are present in *G. segmentatum* sp. nov. and differentiated by 10 nucleotides from *G. multipileum*. Therefore, according to the nucleotide basis, *G. multipileum* is different from our species. *Ganoderma martinicense* Welti and Courtec. basidiospores are larger (9.5–12 × 5–7 μm) with a golden yellow pileal surface (Welti and Courtecuisse, 2010), contrary to *G. segmentatum*. One additional nucleotide is present in *G. martinicense* but absent in our species. Almost seven nucleotides are different in our new species of *G. martinicense*. The spores of *Ganoderma parvulum* are longer (8–10 × 5–6 μm) than those of *G. segmentatum* identified in Pakistan (De Lima Júnior et al., 2014).

*G. parvulum* is characterized by a pale ochraceous context with resinaceous streaks (Murrill, 1902), while the context is light chocolate brown without resinaceous streaks in *G. segmentatum. G. parvulum* has two additional nucleotides, while two gaps are present in our species. Both species are differentiated by 17 nucleotides. The basidiomes of *Ganoderma destructans* and *G. steyaertanum* exhibited a few similarities, but the spores of *G. destructans* are slightly larger than those of *G. steyaertanum* (7.3–12.7 × 5.0–9.5 μm). *G. steyaertanum* possesses laccate dimidiate basidiocarp, occasionally sessile, umbonate that is dark to chestnut (dark brown) in color, yellowish-white margin, and pale yellow to grayish orange pores (Stöver and Müller, 2010). These characteristics are contrary to those of this new *Ganoderma* species. *G. segmentatum* sp. nov. is differentiated from *G. steyaertanum* by eight nucleotides, while our new species possesses one additional nucleotide and two gaps in nucleotide sequence. Basidiospores of *G. destructans* are larger (11–14 × 7–9 μm, av. 12.3 × 8.0 μm) than our new species. The pileus surface of *G. destructans* is covered by white to creamy soft non-poroid tissue of hymenophore, which turns brown when this species becomes old (Coetzee et al., 2015). At the molecular level, analysis of ITS sequence alignments revealed that three additional and five different nucleotides are present in *G. segmentatum* sp. nov., while they are absent in *G. destructans*.

The combinatorial mating system of sexual reproduction promoted diversity in the genus *Ganoderma*. The genetic divergence in the genetic pool was higher, especially in isolates of different geographical origins. Different isolates and strains exhibit a different degree of aggressiveness and patience toward biological control (Kok et al., 2013; Midot et al., 2019; Wong et al., 2021).

In this study, upper and basal stem rot severely affected the *Vachellia nilotica* plant species. This rot is the primary cause of an inefficient disease control system. Studies indicated that there is a need of compatible partners for *Ganoderma* species to “mate and initiate” the sexual cycle (heterothallism). Monokaryons possess two “unlinked mating” loci, which initiate the mating process to complete sexual reproduction (ruled by the tetrapolar mating system) (Ramzi et al., 2019). *Ganoderma*, as a pathogen, releases small cell wall-degrading enzymes, e.g., cellulase, manganese peroxidase, polygalacturonase, and laccase, which soften the host cell wall (Tan et al., 2018). Hyphae of *G. boninense* colonized the oil palm root by secreting trace amounts of degrading enzymes (polygalacturonase and laccase) (Dhillon et al., 2021). These enzymes degenerate the integrity of the polysaccharides (host cell wall) (Ho et al., 2019). This process starts with the production of multiple response molecules (reactive oxygen species, phytoalexins, and pathogenesis-related proteins) to trigger the alterations in the cell wall. Currently, little study is available on the proteins or enzymes involved in host penetration. Hyphal mating ensures the survival and continuation of the genetic variety of many fungal pathogenic species. Variation in mating facilitates the species in adoption of changed environmental conditions (Morrow and Fraser, 2009). Different studies have reported that *Ganoderma* is a common disease-causing agent. This species gradually leads to the death and decline of trees (Glen et al., 2009; Elshafie et al., 2013; Bhadra, 2014; Coetzee et al., 2015). The pathogenic action of *G. adspersum* on young *Tilia* species and *Aesculus* sp. was reported in Italy by Nicolotti et al. (2009). Post-inoculation, after 2 years, make the decay columns by drilling the method of inoculation. In New York, Pirone (1957) also performed the pathogenicity tests via agar plugs or fruiting body (pieces) of *G. lucidum* placed by drilling into the holes of the lower trunk in young *Acer platanoides*, and symptoms of infection were also observed. Elliott and Broschat (2005) considered *Ganoderma zonatum* a destructive palm pathogen, similarly, infections or diseases were observed in palms according to Elliott and Uchida (2024). A few *Ganoderma* species are host-specific and found on certain groups of hosts (palms, conifers, and hardwoods) (Gilbertson and Ryvarden, 1986); For example, *Ganoderma meredithiae* attacked pines, while *Ganoderma curtisii* made connections with hardwoods (Elliott and Broschat, 2005; Adaskaveg et al., 2011).

Non-phenolic substrates are oxidized by white/yellow laccases, which are required in the case of blue laccases. Yellow laccase was detected by converting veratryl alcohol into veratraldehyde (Chaurasia et al., 2013a,b). Veratryl alcohol is a secondary metabolite synthesized *de novo* by white-rot fungi and its low amount enhance the laccase activity (Jensen et al., 1994). Veratryl alcohol, a non-phenolic lignin compound, can be oxidized by the laccase of *Coriolus versicolor*.

In the reported literature on submerged culture, fungi secreted blue laccases, while secreting yellow laccases when grown on lignin-containing solid substrates. Yellow laccases are better biocatalysts than blue ones. Yellow laccase was produced in *Pleurotus ostreatus, G. fornicatum, Panus tigrinus, Phlebia radiate, P. tremellosa, Sclerotinia sclerotiorum* (Daroch et al., 2014; Agrawal et al., 2018), *G. lucidum, Scytalidium thermophilum* (Ben Younes and Sayadi, 2011), *Pycnoporus sanguineus* (Dantán-González et al., 2008), *Cerrena unicolor* (Michniewicz et al., 2008), *Pycnoporus cinnabarinus* (Schliephake et al., 2000), and *Myrothecium verrucaria* (Zhao et al., 2012). The blue laccase was identified by *Trametes versicolor, T. trogii, T. villosa, Rigidoporus lignosus, and Coriolus versicolor* (Levin et al., 2010). *Phellinus linteuis* MTCC-1175 exhibited a peak at 610 nm (Chaurasia et al., 2013a,b), while yellow laccase showed a characteristic spectrum in *Coriolopsis floccosa* MTCC-1177 (Chaurasia et al., 2013a,b).

It was previously observed that the yellow laccases were obtained from cultures grown on a solid-state medium, while the blue forms isolated from cultures grown on a liquid medium without lignin (Daroch et al., 2014). The authors suggested that the yellow laccase can be fabricated by the modification of blue laccase via 1. low-molecular-weight lignin decomposition products; 2. glycosylation; 3. turnover-dependent oxidation of the active site; 4. amino acids; and 5. copper ligands. This mediator can be obtained from the culture medium, from which the enzyme was isolated (Dantán-González et al., 2008; Ben Younes and Sayadi, 2011; Agrawal et al., 2018).

## 5 Conclusion

The morpho-anatomical and molecular study of *Ganoderma segmentatum* indicated that it is a new species matrixed separately in the clade of *Ganoderma* species with a strong bootstrap value (99%). *Ganoderma* is a globally distributed genus comprising species associated with forest ecology and medicinal trees. The *Ganoderma* species are facultative parasites on living, dead, or rotting trees. They also cause the white rot of hardwoods by decomposing cellulose, lignin, and polysaccharides. The decay of roots and lower trunk or stem flares leads to hazardous tree conditions and tree failures, resulting in serious damage to property and life.

It is clear that *Ganoderma* are ecologically indispensable, but their pathogenic nature causes tree diseases. Although this new species is a major contributor to the genus *Ganoderma* and its pathogenic relationship with host plants has brutally damaged the *V. nilotica*.

## Data availability statement

The original contributions presented in the study are included in the article/supplementary material, further inquiries can be directed to the corresponding authors.

## Author contributions

AU: Writing – original draft, Writing – review & editing. WY: Writing – original draft. JL: Writing – review & editing. FA: Writing – original draft.
